# Identification of an N staging system that predicts oncologic outcome in resected left-sided pancreatic cancer

**DOI:** 10.1097/MD.0000000000004035

**Published:** 2016-07-01

**Authors:** Sung Hyun Kim, Ho Kyoung Hwang, Woo Jung Lee, Chang Moo Kang

**Affiliations:** Department of Hepatobiliary and Pancreatic Surgery, Yonsei University College of Medicine, Pancreaticobiliary Cancer Clinic, Yonsei Cancer Center, Severance Hospital, Seoul, Korea.

**Keywords:** lymphatic metastasis, neoplasm staging, pancreatectomy, pancreatic cancer, survival

## Abstract

In this study, we investigated which N staging system was the most accurate at predicting survival in pancreatic cancer patients.

Lymph node (LN) metastasis is known to be one of the important prognostic factors in resected pancreatic cancer. There are several LN evaluation systems to predict oncologic impact.

From January 1992 to December 2014, 77 medical records of patients who underwent radical pancreatectomy for left-sided pancreatic cancer were reviewed retrospectively. Clinicopathologic variables including pN stage, total number of retrieved LNs (N-RLN), lymph node ratio (LNR), and absolute number of LN metastases (N-LNmet) were evaluated. Disease-free survival (DFS) and disease-specific survival (DSS) were analyzed according to these 4 LN staging systems.

In univariate analysis, pN stage (pN0 vs pN1: 17.5 months vs 7.9 months, *P* = 0.001), LNR (<0.08 vs ≥0.08: 17.5 months vs 4.4 months, *P* < 0.001), and N-LNmet (#N = 0 vs #N = 1 vs #N≥2: 17.5 months vs 11.0 months vs 6.4 months, *P* = 0.002) had a significant effect on DFS, whereas the pN stage (pN0 vs pN1: 35.3 months vs 16.7 months, *P* = 0.001), LNR (<0.08 vs ≥0.08: 37.1 months vs 15.0 months, *P* < 0.001), and N-LNmet (#N = 0 vs #N = 1 vs #N≥2: 35.3 months vs 18.4 months vs 16.4 months, *P* = 0.001) had a significant effect on DSS. In multivariate analysis, N-LNmet (#N≥2) was identified as an independent prognostic factor of oncologic outcome (DFS and DSS: Exp (β) = 2.83, *P* = 0.001, and Exp (β) = 3.17, *P* = 0.001, respectively).

Absolute number of lymph node metastases predicted oncologic outcome in resected left-sided pancreatic cancer patients.

## 1 Introduction

Pancreatic cancer is the fifth leading cause of cancer death in the Republic of Korea. Five-year survival rate of pancreatic cancer is <10%.^[1]^ Curative surgery can only be performed in 15% to 20% of patients at the time of diagnosis.^[2]^ Furthermore, even if curative surgery is performed, median survival is poor at 7 to 19 months.^[3]^ Survival after resection is influenced more by tumor-related factors such as tumor size, histologic differentiation, nodal status, and involvement of margins than patient-specific characteristics or surgical techniques.^[4–6]^ Among tumor-related factors, lymph node (LN) metastasis is known to be one of the important prognostic factors regardless of the mechanism of LN involvement.^[7]^

American Joint Committee on Cancer (AJCC) N staging is commonly used, but it describes only the existence of LN metastasis.^[8]^ This staging is too simple to predict oncologic outcome and is not always an independent prognostic factor.^[9]^ Therefore, many physicians have attempted to develop alternative LN evaluation systems to predict pancreatic cancer outcome. Total number of retrieved LNs (N-RLN), lymph node ratio (LNR), and absolute number of LNs metastases (N-LNmet) have been reported as alternative LN evaluation systems.^[10–16]^ However, these systems have some limitations with regard to clearly predicting oncologic outcome. For example, the N-RLN system is not accurate in node-positive pancreatic cancer patients.^[10–12]^ In contrast, the LNR system is not accurate in node-negative pancreatic cancer patients.^[12,13]^ Furthermore, 2 studies reported no linear correlation between the absolute number of LNs metastases and the survival rate using the N-LNmet system.^[13,16]^

In this study, we investigated which N staging system among those listed above could accurately predict survival differences in resected left-sided pancreatic cancer patients.

## 2 Methods

From January 1992 to December 2014, 117 medical records of patients who underwent radical pancreatectomy for left-sided pancreatic cancer were reviewed retrospectively. In total, 31 patients who underwent pancreatectomy following neoadjuvant treatment were excluded and 9 patients who were revealed R1 resection at the pathologic report were also excluded. Following distal pancreatectomy, almost all patients received adjuvant chemotherapy, except for those patients who had poor performance status or those who refused adjuvant chemotherapy. A total of 77 patients who underwent curative resection were included in the analyses. Clinicopathologic variables including pN stage, N-RLN, LNR, and N-LNmet were evaluated. Disease-specific survival (DSS) and disease-free survival (DFS) were analyzed according to these 4 different lymph node staging systems.

IBM SPSS Statistics version 20.0 (IBM Corp., Somers, NY) was used for all statistical analyses. Nominal data were compared with χ^2^ tests and continuous data with *t* tests. Survival parameters were assessed by the Kaplan–Meier method and compared with the log-rank test. A Cox proportional hazards model was used for multivariate survival analysis. Variables with *P* < 0.05 after univariate analysis were used in the multivariate analysis. This study was approved by our institutional review board (IRB No. 4-2015-0296).

## 3 Results

### 3.1 Patients’ characteristics

The clinicopathologic characteristics of the 77 patients who underwent radical pancreatectomy for left-sided pancreatic cancer are shown in Table 1. According to the AJCC pN staging system, 38 patients (49.4%) were pN0 and 39 patients (50.6%) were pN1. Thirty-two patients (41.6%) were N-RLN < 12, and 45 patients (58.4%) were N-RLN ≥12. Forty-six patients (61.3%) were LNR <0.08 and 29 patients (38.7%) were LNR ≥ 0.08. Thirty-eight patients (49.4%) were N-LNmet stage 0 (N# = 0), 17 patients were #N = 1 (22.1%), and 22 patients (28.6%) were #N≥2.

**Table 1:**
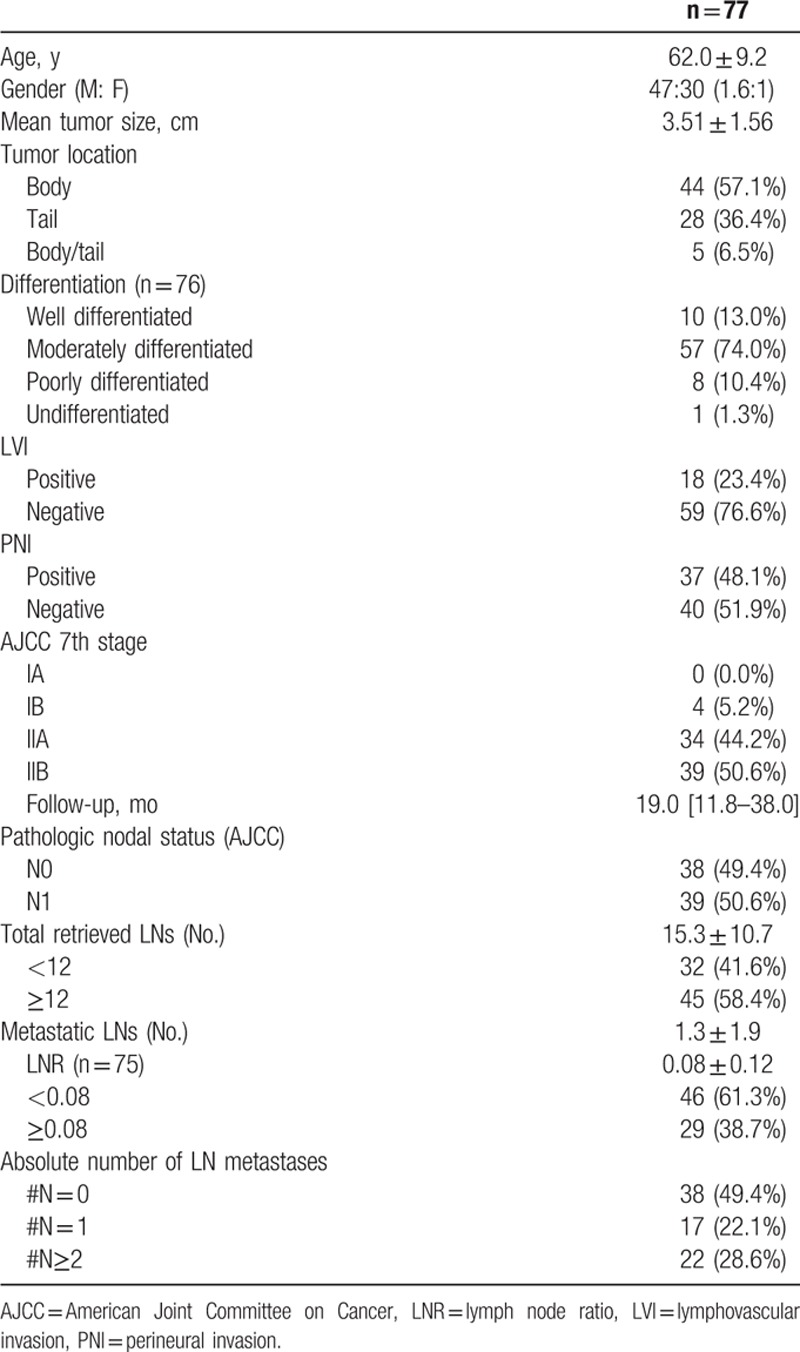
Clinicopathologic characteristics.

### 3.2 Pathologic characteristics according to the LN evaluation system

There were no significant differences in the clinicopathological characteristics of tumor size, differentiation, R0 resection, T stage, perineural invasion, and lymphovascular invasion according to the LN evaluation system (pN stage, N-RLN, LNR, and N-LNmet) (Table 2).

**Table 2:**
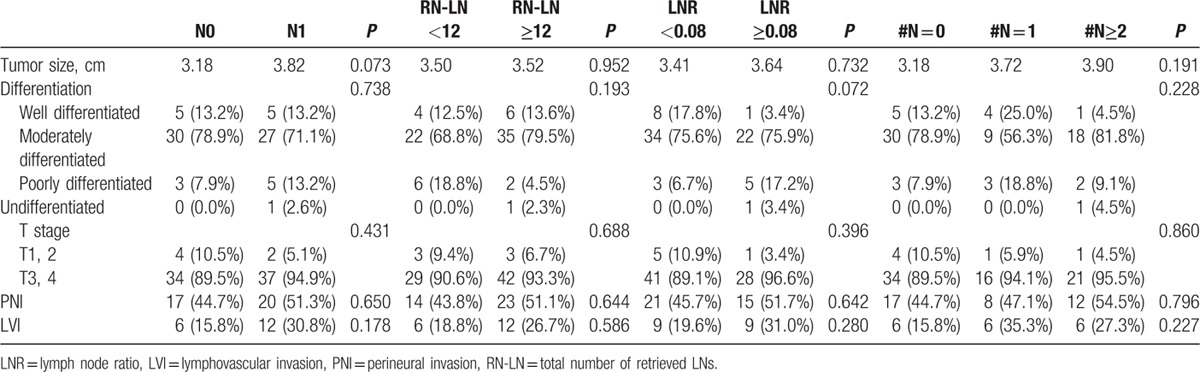
Pathologic characteristics according to the LN evaluation system after curative resection.

### 3.3 Oncologic outcomes according to the LN evaluation system

**Figure 1:**
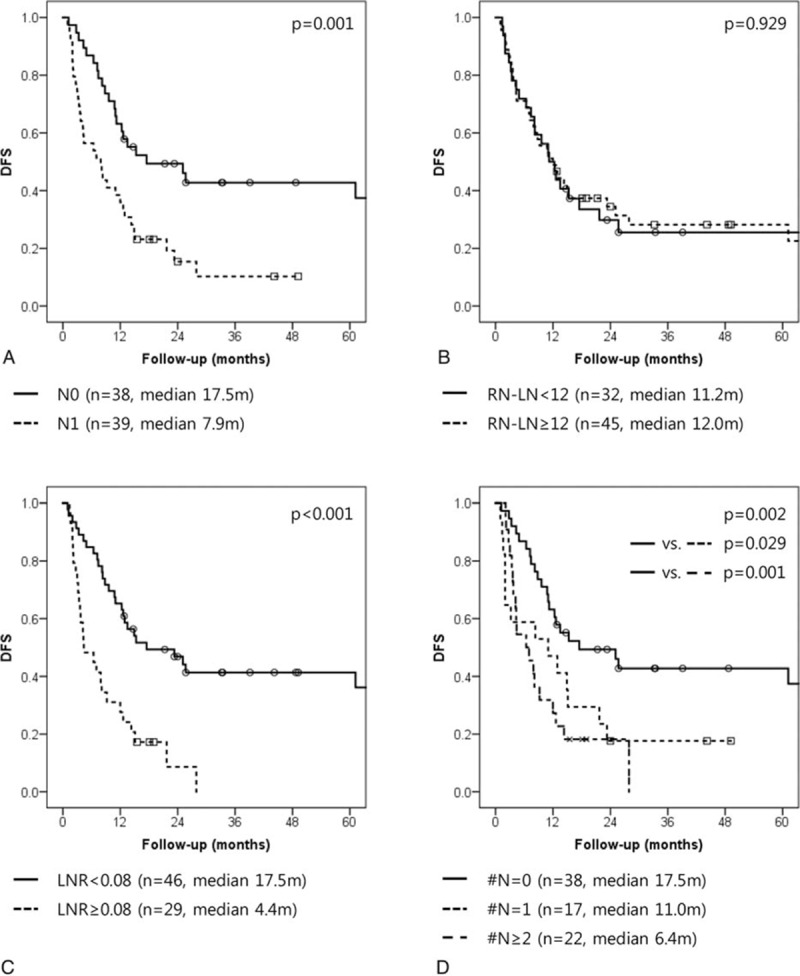
Disease-free survival rate according to LN evaluation system. (A) Pathologic N stage (pN), (B) total number of retrieved LNs (RN-LN), (C) lymph node ratio (LNR), (D) absolute number of LN metastases (N-LNmet). LN = lymph node, LNR = lymph node ratio, N-LNmet = absolute number of LN metastases, pN = pathologic N stage, RN-LN = total number of retrieved LNs.

The median overall DFS was 12.0 months (95%CI: 8.9–15.1), and the median DSS was 21.4 months (95%CI: 14.1–28.7). In univariate analysis, the pN stage (N0 vs N1: 17.5 months [95%CI: 1.6–33.4] vs 7.9 months [95%CI: 3.1–12.7], *P* = 0.001), N-RLN (<12 vs ≥12: 11.2 months [95%CI: 7.0–15.4] vs 12.0 months [95%CI: 7.2–16.8], *P* = 0.929), LNR (<0.08 vs ≥0.08: 17.5 months [95%CI: 3.7–31.3] vs 4.4 months [95%CI: 0.5–8.3], *P* < 0.001), and N-LNmet (#N = 0 vs #N = 1 vs #N≥2: 17.5 months [95%CI: 1.6–33.4] vs 11.0 months [95%CI: 0.0–24.0] vs 6.4 months [95%CI: 2.3–10.5], *P* = 0.002) had a significant effect on DFS (Fig. 1).

**Figure 2:**
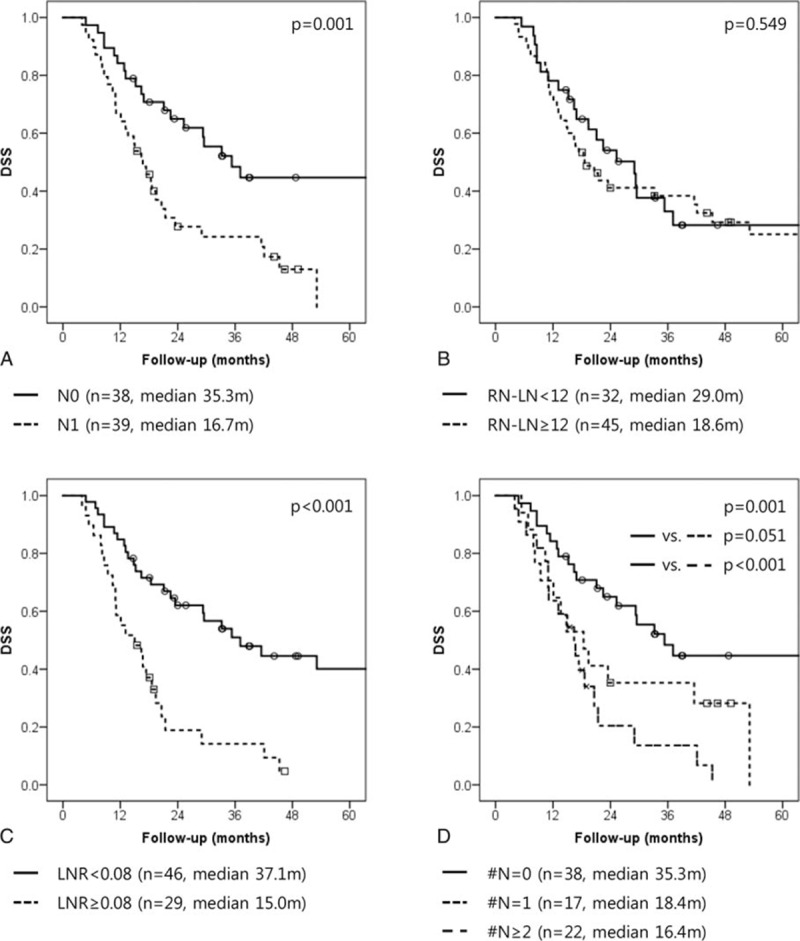
Disease-specific survival rate according to the LN evaluation system. (A) Pathologic N stage (pN), (B) total number of retrieved LNs (RN-LN), (C) lymph node ratio (LNR), (D) absolute number of LN metastases (N-LNmet). LN = lymph node, LNR = lymph node ratio, N-LNmet = absolute number of LN metastases, pN = pathologic N stage, RN-LN = total number of retrieved LNs.

In addition, these LN evaluation systems were also found to influence DSS (pN0 vs pN1: 35.3 months [95%CI: 23.2–47.4] vs 16.7 months [95%CI: 12.5–20.9], *P* = 0.001); (RN-LN<12 vs ≥12: 29.0 months [95%CI: 19.5–38.5] vs 18.6 months [95%CI: 12.8–24.4], *P* = 0.549); (LNR<0.08 vs ≥0.08: 37.1 months [95%CI: 21.7–52.5] vs 15.0 months [95%CI: 7.6–22.4], *P* <0.001); (N-LNmet #N = 0 vs #N = 1 vs #N≥2: 35.3 months [95%CI: 23.2–47.4] vs 18.4 months [95%CI: 10.6–26.2] vs 16.4 months [95%CI: 11.2–21.6], *P* = 0.001) (Fig. 2).

In multivariate analysis, N-LNmet was identified as an independent prognostic factor of oncologic outcome in resected left-sided pancreatic cancer patients (#N = 1: DFS and DSS: Exp (β) = 2.17 [95%CI: 1.10–4.29], *P* = 0.025, and Exp (β) = 2.04 [95%CI: 1.01–4.15], *P* = 0.049, respectively); (#N≥2: DFS and DSS: Exp (β) = 2.83 [95%CI: 1.50–5.36], *P* = 0.001, and Exp (β) = 3.17 [95%CI: 1.64–6.13], *P* = 0.001, respectively). In contrast, LNR was not identified as an independent prognostic factor (DFS and DSS: Exp (β) = 2.94 [95%CI: 0.95–9.12], *P* = 0.062, and Exp (β) = 2.89 [95%CI: 0.82–9.58], *P* = 0.098) (Table 3).

**Table 3:**
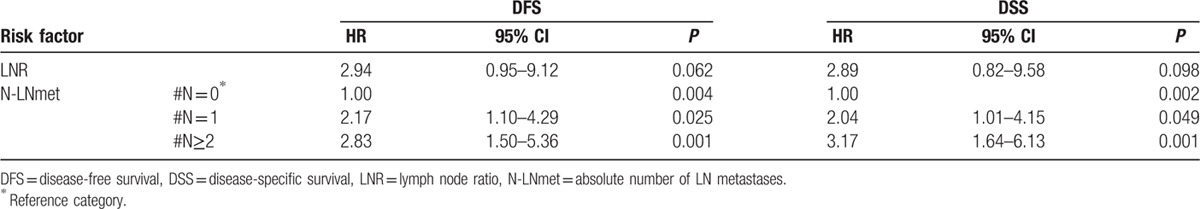
Hazard ratio for risk factors in patients.

### 3.4 Oncologic outcomes according to adjuvant chemotherapy based on N-LNmet staging (#N = 1)

**Figure 3:**
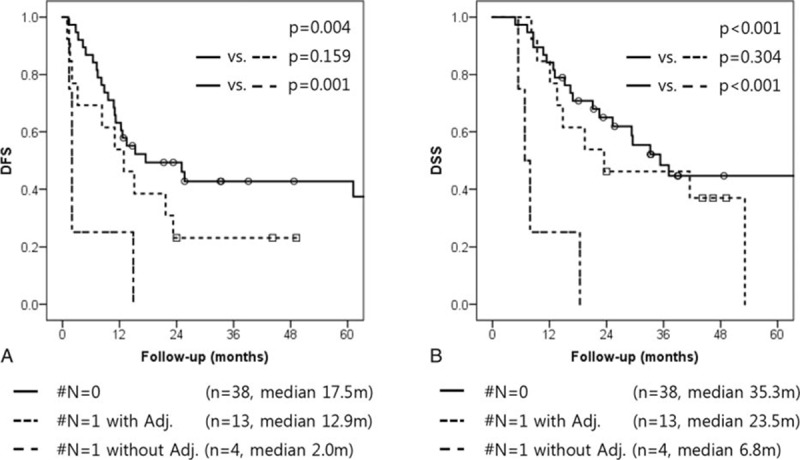
Survival rate according to adjuvant therapy in N-LNmets #N = 1 group. (A) Disease-free survival; (B) disease-specific survival. N-LNmet = absolute number of LN metastases.

There was a significant difference in DFS and DSS among N-LNmet stage 0 (#N = 0) patients, N-LNmet stage 1 (#N = 1) patients treated with adjuvant chemotherapy (Adj-CTx) and N-LNmet #N = 1 patients treated without adjuvant chemotherapy (DFS: #N = 0 vs #N = 1 with Adj-CTx. vs #N = 1 without Adj-CTx: 17.5 months [95%CI: 1.6–33.4] vs 12.9 months [95%CI: 4.9–20.9] vs 2.0 months [95%CI: 1.5–2.5], *P* = 0.004); (DSS: N-LNmet #N = 0 vs #N = 1 with Adj. vs #N = 1 without Adj.: 35.3 months [95%CI: 23.2–47.4] vs 23.5 months [95%CI: 0.0–52.9] vs 6.8 months [95%CI: 4.4–9.3], *P* < 0.001) (Fig. 3).

### 3.5 Comparable oncologic impact of N-LNmet (#N≥2) with unresected left-sided pancreatic cancer

**Figure 4:**
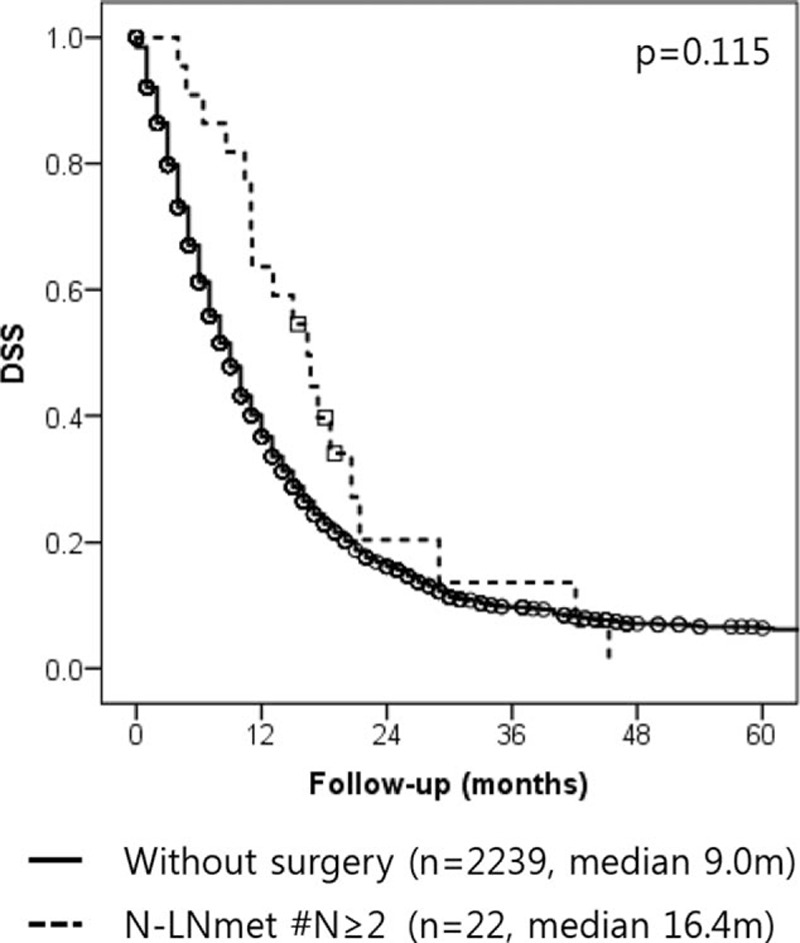
Disease-specific survival rate of without surgery group and N-LNmets #N≥2 group. N-LNmet = absolute number of LN metastases.

Among those patients who underwent resection of left-sided pancreatic cancer, patients with #N≥2 showed comparable survival to those who did not undergo surgical resection (n = 2239, from 2005–2014) (#N≥2 vs nonsurgical resection: 16.4 months [95%CI: 11.2–21.6] vs 9.0 months [95%CI: 8.5–9.5], *P* = 0.115) (Fig. 4).

## 4 Discussion

Much effort has been invested in finding LN evaluation systems that accurately predict prognosis in pancreatic cancer patients. The more LNs that are retrieved, the greater the likelihood of an accurate prognosis.^[13]^ Several studies have reported results from thorough harvesting of LNs during pancreatectomy. These studies reported significant cut-off values based on 10 to 15 total retrieved LNs.^[10–13,17–19]^ However, an adequate number of LNs are sometimes not retrieved by standard lymphadenectomy, especially in those patients who have undergone neoadjuvant chemotherapy.^[20]^ We argue that because extended lymphadenectomy has not been proven to improve oncologic outcome to our knowledge, this surgery should not be performed solely to predict prognosis.^[17,21]^ Moreover, the total number of LNs examined is dependent on not only the surgeons, but also the pathologists who examine the surgical specimen.^[10,12,13,17,18]^ These factors can result in “stage migration,” which can in turn influence prognosis.^[22]^

LNR may be prognostic in patients with pancreatic cancer.^[12–15,19,23,24]^ Reported LNR cut-off values range from 0.1 to 0.4.^[12,13,19,23,24]^ However, regardless of the number of retrieved LNs, the LNR of pathologically node-negative patients is 0, which does not allow clear prediction of prognosis. In addition, LNR can present with a stratified pattern due to the influence of LN metastasis. Therefore, unlike retrieved LN studies, various cut-off values have been reported according to the characteristics of the cohort of patients examined. To address these limitations, log odds of positive lymph nodes (LODDS) and Bayesian models have been proposed.^[14,15]^ However, these evaluation systems are complex to apply. In addition, these systems are also based on LNR, which as mentioned previously, has limited prognostic ability in the node negative patient group.

Number of metastatic lymph nodes (N-LNmet) is another established prognostic factor in pancreatic cancer.^[13,16,23,25,26]^ The greater the number of LN metastases, the worse the prognosis of the patient is expected to be. However, at the beginning set point, the survival rate of patients decreased rapidly in our study. Set points in our study varied from 2 to 5; similar patterns have been reported in other studies.^[13,16,23,25,26]^ We found that the #N≥2 group of patients had a different survival pattern than the other groups. More than 2 metastatic lymph nodes were found to be independent prognostic factor of early recurrence and long-term survival (Figs. 1 and 2). In particular, the #N≥2 group showed a similar DFS to that of patients who did not undergo surgical resection (Fig. 4). Moreover, in #N = 1 subgroup analysis, #N = 1 patients who did not undergo adjuvant chemotherapy had an inferior prognosis to those who underwent adjuvant chemotherapy (Fig. 3). This result suggests that severe LN metastasis might attenuate the oncologic effect of radical pancreatectomy and neoadjuvant therapy; primary surgical resection may be a better option in this group. In other words, the application of appropriate adjuvant treatment modalities in those patients with minimal LN metastasis, like the #N = 1 group, might result in a survival rate similar to that of node-negative patients.

Note that in this study, all patients underwent radical pancreatectomy for left-sided pancreatic cancer. Distinction between periampullary and other pancreatic adenocarcinoma is not always easy; expert pathologists often have difficulty distinguishing these 2 cancer types. Consequently, the pathologic diagnosis is changed by other pathologists once in a while.^[27]^ Moreover, some studies have reported that only half of pancreatic cancer patients who survived >5 years had original pancreatic head cancer slide based on re-evaluation of the original slides.^[28,29]^ These misdiagnoses can affect the survival rate of specific diseases and complicate analyses. To avoid this, we confined disease to left sided-pancreatic cancer.

Several studies have revealed that there are differences in lymph node streams according to the type of pancreatic cancer; it is assumed that pancreas head cancer and left-sided pancreatic cancer have unique biological characteristics. Some physicians have therefore suggested that these cancers might benefit from different treatment approaches.^[26,30–33]^ We focused on left-sided pancreatic cancer and found that 1 of the 4 LN evaluation systems we evaluated predicted prognosis accurately in multivariate analysis.

Limitations of this study include its retrospective study design. Because of uncontrolled adjuvant therapy, we did not analyze relevance according to chemotherapy regimens. We also did not analyze relevance of lymph node area as prognostic value due to unclearness of the records of dissected lymph node areas. However, we provided insight into prognostic factors for left-sided pancreatic cancer. Multivariate analysis was used to address some of the limitations of our study design and revealed that N-LNmet was a more significant predictor of oncologic outcome than LNR. In conclusion, we found that N-LNmet as an independent staging system accurately predicted DFS and DSS in patients with left-sided pancreatic cancer. Patients with #N≥2 showed a poor survival rate, suggesting that these patients are potential candidates for neoadjuvant treatment. Further studies to identify this patient group preoperatively are warranted.
